# Sleep during COVID‐19‐related school lockdown, a longitudinal study among high school students

**DOI:** 10.1111/jsr.13499

**Published:** 2021-10-03

**Authors:** Ingvild West Saxvig, Ståle Pallesen, Børge Sivertsen, Mari Hysing, Linn Nyjordet Evanger, Bjørn Bjorvatn

**Affiliations:** ^1^ Norwegian Competence Center for Sleep Disorders Haukeland University Hospital Bergen Norway; ^2^ Centre for Sleep Medicine Haukeland University Hospital Bergen Norway; ^3^ Department of Psychosocial Science University of Bergen Bergen Norway; ^4^ Optentia Research Focus Area North‐West University, Vanderbijlpark Campus Vanderbijlpark South Africa; ^5^ Department of Health Promotion Norwegian Institute of Public Health Bergen Norway; ^6^ Department of Research and Innovation Helse Fonna HF Haugesund Norway; ^7^ Department of Mental Health Norwegian University of Science and Technology Bergen Norway; ^8^ Department of Global Public Health and Primary Care University of Bergen Bergen Norway

**Keywords:** adolescents, circadian typology, COVID‐19, school lockdown, sleep

## Abstract

There has been great concern about the impact of coronavirus disease 2019 (COVID‐19)‐related school lockdown on adolescent health. The aim of the present study was to compare sleep patterns before and during COVID‐19‐related school lockdown, in a large sample of high school students. The present study is based a prospective, longitudinal survey on adolescent sleep health. Phase 1 was conducted in 2019, whereas phase 2 was conducted in 2020 (response rate 60.2%), during the last 10 days of a 60‐day long school lockdown. Main outcomes comprised sleep parameters from the Munich ChronoType Questionnaire (MCTQ). A total of 2,022 students provided valid responses to MCTQ in both survey phases. Results showed later sleep timing on schooldays in 2020 compared to 2019 (36 min later bedtimes, Cohen’s *d* = 0.56; 1:35 hr later rise times, Cohen’s *d* = 1.44). Time spent in bed on schooldays increased from 8:20 to 9:19 hr (Cohen’s *d* = 0.78), and sleep duration increased by 45 min (Cohen’s *d* = 0.49). The proportion of adolescents obtaining the recommended ≥8 hr of sleep on schooldays increased from 13.4% (2019) to 37.5% during the lockdown. Social jetlag was reduced from 2:37 hr (2019) to 1:53 hr (2020, Cohen’s *d* = 0.59). Results points to a potential advantageous effect of school lockdown in terms of increased school day sleep duration and reduced social jetlag. As sleep is important for mental and somatic health, it is conceivable that increased sleep duration offered some protection against harmful aspects of the COVID‐19 pandemic and associated social restrictions. Future studies should address possible associations between sleep changes and health during COVID‐19‐related school lockdown.

## 1 INTRODUCTION

As a response to the public health threat posed by the coronavirus disease 2019 (COVID‐19) pandemic, governments across the globe have attempted to reduce the spread of the severe acute respiratory syndrome coronavirus‐2 (SARS‐CoV‐2) by implementing measures to restrict gathering and movement of people. Although the details of such restrictions have varied across countries and pandemic phases, most nations maintained school lockdown/remote teaching during the initial phase of the pandemic (spring 2020).

Paediatric clinicians and researchers have been greatly concerned about possible harmful effects of the COVID‐19 pandemic and associated social restrictions on child and adolescent mental health, in particular with respect to school lockdown (Golberstein et al., 2020). As school normally plays an important role in the lives of many adolescents, by providing daily routines and social support, concerns have been raised that school lockdown may not only add to the mental burden of the pandemic, but also restrict access to an important arena for intervention and care (Golberstein et al., 2020). In line with such notions, studies have indicated an increase in sleep and mental health problems among adolescents during the pandemic (Bhatia, 2020; Magson et al., 2021; Nearchou et al., 2020; Zhou et al., 2020).

Still, it has been suggested that, in terms of sleep, some adolescents may in fact benefit from the situation. In a recent editorial in *Journal of Child Psychology and Psychiatry*, Becker and Gregory recognise that adolescents may be particularly vulnerable to social isolation and disruption of daily routines, but that, at the same time, some may benefit from reduced social time pressure, especially related to school lockdown/remote teaching (Becker & Gregory, 2020). Adolescence is generally characterised by a drift towards eveningness, reflecting a late biological temporal preference for sleep and activity (Randler et al., 2016, 2017). In an interplay with early morning obligations (e.g. early school start times), adolescents normally have short school day sleep duration (late bed times combined with early rise times), as well as a large social jetlag (difference between midsleep on school/work days and free days) (Carskadon, 2011; Crowley et al., 2018; Gariepy et al., 2020; Saxvig et al., 2020). Further, evening types (those with most eveningness/latest temporal preference for sleep) have been found to have the shortest school day sleep duration and largest social jetlag compared the other circadian types (intermediate and morning types) (Adan et al., 2012; Roenneberg et al., 2007; Saxvig et al., 2021). Against this backdrop, it seems plausible that school lockdown may allow adolescent high school students in general, and evening types in particular, more opportunity to sleep at times that are better aligned with each person’s biological timing for sleep.

In accordance with this notion, a recent prospective study conducted among 122 adolescents, demonstrated not only increased difficulties initiating sleep, but also longer school day sleep duration during the COVID‐19 pandemic (Becker et al., 2021). Also studies in older and more diverse samples have shown increased sleep duration and reduced social jetlag during the COVID‐19 pandemic, with the largest changes seen in younger people (Korman et al., 2020; Leone et al., 2020; Wright et al., 2020). Still, large scale prospective studies specifically addressing the impact of COVID‐19 on sleep among adolescent high school students, the group who is likely to benefit the most from reduced social time pressure, are lacking from the literature. Furthermore, to our knowledge, no studies have to date addressed the effects of school lockdown on adolescent sleep in relation to circadian typology.

The aim of the present study was to compare adolescent sleep patterns between pre‐pandemic times (spring 2019) and the initial phase of the COVID‐19 pandemic when schools were in lockdown (spring 2020), overall and in relation to day (schooldays versus free days) and circadian typology (morning types versus intermediate types versus evening types), based on data from a longitudinal survey on sleep health in Norwegian high school students (the Western Norway Adolescent Longitudinal Sleep Study [WALOSS]). We hypothesised that school day sleep would be later and longer, and that social jetlag would be reduced during the lockdown as compared to spring 2019. We further hypothesised that these changes would be more pronounced in evening types compared to the other circadian types.

## 2 METHODS

### 2.1 Procedures

In spring 2019, all first year high school students aged ≥16 years in Hordaland and Rogaland counties in Norway were invited to participate in the WALOSS. The initial phase was facilitated by the county school authorities (schools were encouraged to allocate one school hour to respond to the questionnaire) and had a response rate of 42% (11,574 invited, 4,863 responders). Detailed procedures for phase 1 has previously been described elsewhere (Saxvig et al., 2020). The WALOSS cohort comprised the 3,736 responders who also consented to data linkage with the county school authorities. For phase 2, invitations were sent through Short Message Service (SMS) and private email, and participants were required to respond to the questionnaire in their spare time. As an incentive to participate, all who completed phase 2 took part in a lottery where the price was five iPhone 11 Pros and 100 gift cards, each with a value of 500 Norwegian Kroner (NOK), ~$60 (American dollars). A total of 2,249 students responded in phase 2, yielding a response rate of 60.2%. The survey was technically administered by SurveyXact (Rambøll Management Consulting AS, www.surveyxact.no). The study was funded through a postdoctoral grant provided by the Western Norway Regional Health Authority (Helse Vest RHF). The funding source had no other involvement in the study.

### 2.2 Settings

Phase 1 was conducted from April 10 through to June 20, 2019, whereas phase 2 was conducted from April 30 through to May 10, 2020, which represent the last 10 days of a 60‐day long lockdown of Norwegian high schools (March 12 through to May 10). During the school lockdown period, students were required to attend remote classes, by means of digital media. Other coronavirus mitigation measures during this period included travelling restrictions and closure of arenas for sports, culture and recreation, and the population was encouraged to practice social distancing.

### 2.3 Sample

With respect to the present study, 3,696 students (98.9% of the cohort) completed the relevant sleep questionnaire (the Munich ChronoType Questionnaire [MCTQ], see later) in phase 1, whereas 2,042 of these (55.2%) completed MCTQ in phase 2 (44.8% did not complete MCTQ in phase 2 and were considered non‐responders). Prior to analyses we excluded data from 20 responders (and 27 non‐responders) with obvious invalid responses (negative sleep duration on either schooldays or free days in either phase 1 or phase 2), thus the final sample comprised 2,022 high school students.

### 2.4 Ethics

The study was approved by the Regional Committee for Medical and Health Research Ethics (REK sør‐øst 2019/110) and the Norwegian Centre for Research Data (NSD number 758174). All participants provided informed consent to participate in the study. According to Norwegian regulations, individuals aged ≥16 years are regarded as competent regarding the decision to part take in research.

### 2.5 Instruments

#### 2.5.1 Background information

The survey included items on date of birth, sex and parental education, and students were asked to report their normal school start times (phase 1) or start time for remote teaching (phase 2). In phase 2 they were asked if they were still high school students.

#### 2.5.2 Sleep

The sleep habits questionnaire included the MCTQ (Roenneberg et al., 2003), assessing habitual sleep for schooldays and free days separately. The Norwegian version of the MCTQ was adapted for children/adolescents for the purpose of the present study, based on the English version of the MCTQ for children/adolescents (www.thewep.org). In addition, the questionnaire included an item on wakefulness during the sleep period (WASO): “For how long are you awake during the night on schooldays/free days?”. All responses were provided on drop‐down menus, time items on 15‐min interval scales and latency/duration items on 5‐min interval scales. In the present study, we report bedtime, rise time, time spent in bed (interval from bedtime to rise time) and sleep duration (time in bed minus wakefulness in bed), for schooldays and free days, respectively, as well as social jetlag (calculated as the midpoint of sleep on free days [MSF] minus the midpoint of sleep on schooldays). For more details on how these parameters were computed, see Saxvig et al. (2020). In addition, as a measure of biological timing for sleep (chronotype), we calculated sleep corrected MSF (MSFsc) according to the formula: *MSFsc = MSF − (sleep period on free days* *– average weekly sleep period)/2* (Roenneberg et al., 2019). The MSFsc was estimated only for those who did not use an alarm clock on free days, and when the sleep period on free days exceeded the sleep period on schooldays, see www.thewep.org for detailed description of the computation. Based on the recommendations by the USA National Sleep Foundation, sleep duration on school days was categorised into ≥8 hr (recommended), 7–8 hr (may be appropriate), and <7 hr (not recommended) (Hirshkowitz et al., 2015). Further, in order to visualise sleep duration in relation to circadian typology, the adolescents were grouped into seven categories based on their sleep duration: <5, 5–6, 6–7, 7–8, 8–9, 9–10 and ≥10 hr.

#### 2.5.3 Circadian typology

Circadian typology was measured using the short version of the Horne‐Ostberg Morningness–Eveningness Questionnaire (rMEQ) (Adan & Almirall, 1991; Horne & Ostberg, 1976). The rMEQ is a brief five‐item questionnaire on circadian preference that has been shown to correlate well with other measures of circadian typology (Di Milia et al., 2013). Scores range from 4 to 26, with higher scores indicating more morningness. For the present study, we used rMEQ data completed in phase 1 to classify each respondent as either morning, intermediate, or evening types based on the following cut‐offs: morning types >17, intermediate types 12–17, and evening types <12 (Adan & Almirall, 1991; Danielsson et al., 2019). Cronbach’s alpha for rMEQ was 0.54 in the present study and the mean inter‐item correlation was 0.21.

### 2.6 Statistics

The IBM Statistical Package for the Social Sciences (SPSS®) version 25 (IBM Corp., Armonk, NY, USA) was used for statistical analyses. To address possible selection bias, background information (age, sex, maternal and paternal education) as well the main outcome measures (circadian typology, sleep duration, and social jetlag) were compared between respondents and non‐respondents, using *t* tests for independent samples and chi‐square analyses. Within the final sample, self‐reported school start times in phase 1 were compared to start time for remote classes in phase 2 using paired samples *t* tests. Alpha was set to 0.05.

Three‐way analyses of variance (ANOVAs; year × day × type) were conducted for bedtime, rise time, time in bed and sleep duration to address overall differences in relation to year (2019 versus 2020), day (school day versus free day) and circadian type (morning types versus intermediate types versus evening types), whereas two‐way ANOVAs (year × type) were conducted for social jetlag and chronotype. Alpha was set to 0.008 to implement Bonferroni correction for the six sleep parameters analysed (0.05/6). Significant year × day interaction effects were further explored using paired samples *t* tests (2019 versus 2020) for schooldays and free days separately, and the magnitude of change was explored by calculating Cohen’s *d* for paired samples, with values of 0.2 considered small, 0.5 considered moderate, and 0.8 considered large effect sizes, respectively (Cohen, 1988). Significant year × day × type interaction effects (year × type interaction effect for social jetlag and chronotype) were explored by comparing the mean change for each parameter (ΔSleep = Sleep 2020 – Sleep 2019) using Tukey honestly significant difference (HSD) post hoc tests. Chi‐square analyses were used to compare the proportion of adolescents obtaining <7, 7–8 and ≥8 hr of sleep on schooldays in 2019 and 2020, respectively.

## 3 RESULTS

The mean (*SD*) age in the sample, when responding to phase 1, was 16.5 (1.1) years, 58.3% were female (41.7% male), and 55.2% had mothers/45.2% had fathers with university/college education. All were high school students in 2019, and most (98.2%) reported that they were still high school students in 2020. The mean self‐reported school start time in 2019 was 8:13 a.m. (± 18 min), whereas self‐reported start times for remote classes in 2020 was 8:36 a.m. (± 48 min) (*p* < 0.001, Cohen’s *d* = 0.70). The distribution of circadian typology was 9.7% morning, 53.2% intermediate, and 37.1% evening types.

Compared to the responders, non‐responders were slightly older (0.3 years difference, *p* < 0.001) and more often male (9.1% difference, *p* < 0.001), and fewer had mothers with university/college education (3.4% difference, *p* < 0.039). There was no difference in paternal education between non‐responders and responders (*p* = 0.061). For the baseline measures (phase 1), non‐responders were more often evening types (3.7% difference, *p* = 0.022), had shorter school day sleep duration (12 min difference, *p* < 0.001) and longer social jetlag (10 min difference, *p* < 0.001) than the responders, whereas free day sleep duration did not differ between these groups (*p* = 0.439).

**FIGURE 1:**
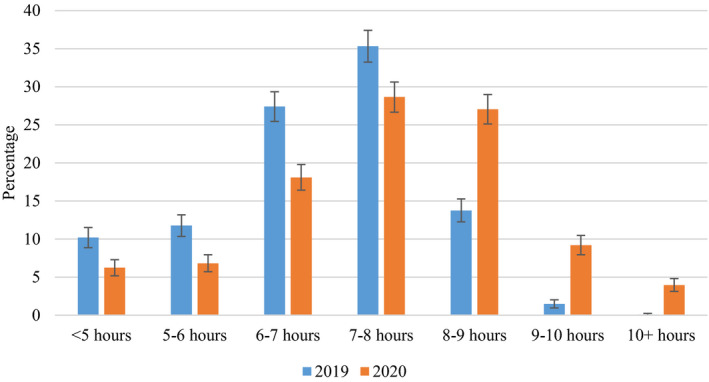
The proportion of high school students obtaining <5, 5–6, 6–7, 7–8, 8–9, 9–10 and 10+ h of sleep in 2019 and 2020, respectively. Error bars denote 95% CI [Colour figure can be viewed at wileyonlinelibrary.com]

Results from the three‐way ANOVAs showed main effects of year as well as year × day interaction effects for all sleep parameters (bedtime, rise time, time in bed, sleep duration) (Table 1). On schooldays, the mean bedtime was 36 min later (moderate effect size) and rise time 1:34 hr later (large effect size) in 2020 compared to 2019 (Table 1). The mean school day rise time in 2020 was as late as 8:25 a.m. (Table 1), which is only 11 min earlier than mean self‐reported start time for remote teaching. Reflecting the combined changes in bedtime and rise time, the adolescents spent almost an hour longer in bed on schooldays (59 min) in 2020 compared to 2019 (large effect size), and sleep duration was 45 min longer (moderate effect size) (Table 1). The proportion of adolescents who obtained the recommended ≥8 hr of sleep on schooldays increased from 13.4% in 2019 to 37.5% in 2020, whereas the proportion who obtained <7 hr of sleep decreased from 49.4% in 2019 to 31.2% in 2020 (37.3% obtained 7–8 hr of sleep in 2019 compared to 31.3% in 2020), *χ* ^2^(4) = 226.79, *p* < 0.001. School day sleep durations in 2019 and 2020 are illustrated in Figure 1. On free days, the mean bedtime was only 9 min later and rise time only 11 min later in 2020 compared to 2019 (small effect sizes) (Table 1), and time in bed and sleep duration did not change from 2019 to 2020 (Table 1).

**TABLE 1:** Sleep characteristics in adolescents before and during the COVID‐19‐related school lockdown (2019 versus 2020), *N* = 2,022

	Sleep characteristics				ANOVA^b^							
	2019	2020		*d* ^a^	Year		Year × type		Year × day		Year × day × type	
					*F*	*p*	*F*	*p*	*F*	*p*	*F*	*p*
Bedtime, hr:min ± min												
Schooldays	22:30 ± 52	23:06 ± 76	*	0.56	149.87	<0.001	0.946	0.388	120.58	<0.001	3.02	0.049
Free day	00:24 ± 80	00:34 ± 89	*	0.12								
Rise time, hr:min ± min												
Schooldays	06:50 ± 35	08:25 ± 97	*	1.44	543.29	<0.001	5.62	0.004	525.52	<0.001	40.58	<0.001
Free day	11:00 ± 100	11:12 ± 106	*	0.12								
Time in bed, hr:min ± min												
Schooldays	08:19 ± 55	09:18 ± 97	*	0.78	127.10	<0.001	4.16	0.016	216.59	<0.001	24.15	<0.001
Free day	10:35 ± 97	10:37 ± 100		0.02								
Sleep duration, hr:min ± min												
Schooldays	06:43 ± 87	07:28 ± 98	*	0.49	61.22	<0.001	3.03	0.048	144.95	<0.001	19.94	<0.001
Free day	08:35 ± 97	08:36 ± 92		0.01								
Social jetlag, hr:min ± min	02:37 ± 65	01:53 ± 70	*	0.59	369.98	<0.001	24.31	<0.001				
Chronotype, hr:min ± min^c^	04:56 ± 75	05:08 ± 86		0.15	8.88	0.003	6.21	0.002				

All values are presented as mean ± *SD*.

^a^ Cohen’s *d* for paired samples, as a measure of effect size (magnitude of change).

^b^ Results from overall ANOVAs in relation to year (2019 versus 2020), day (school day/free day) and circadian type (morning types versus intermediate types versus evening types). Bedtime, rise time, time in bed, and sleep duration were analysed using three‐way ANOVAs (day × year × type), whereas social jetlag was analysed using two‐way ANOVAs (year × type). *F* and *p*‐values are reported for main effect of year and interaction effects of year × type, year × day, and year × day × type. Alpha was set to 0.008.

^c^ Chronotype (MSFsc) could only be calculated for those who did not use an alarm clock on free days, hence *n* = 1,328 for this analysis.

* Significant differences from 2019 to 2020 by paired samples *t* tests on schooldays and free days separately (bedtime, rise time, time in bed, sleep duration) or significant effect of year in the overall two‐way ANOVA (social jetlag). Alpha was set to 0.008.

Mirroring the large changes in school day sleep combined with small changes in free day sleep from 2019 to 2020, we found a significant effect of year for social jetlag, which was reduced by 44 min in 2020 as compared to 2019 (moderate effect size) (Table 1). We also found a significant effect of year for chronotype (calculated for 1,328 adolescents), but the delay was only 12 min (small effect size) (Table 1).

**FIGURE 2:**
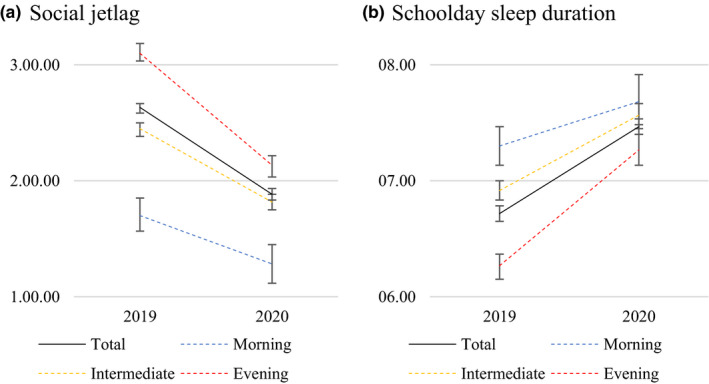
Changes in social jetlag (a) and schoolday sleep duration (b) from 2019 (pre‐pandemic) to 2020 (during COVID‐19 related school lockdown), overall and in relation to circadian typology. Error bars denote 95% CI, further statistics are displayed in Tables 1 and 2

Addressing the role of circadian typology, results from the two‐way ANOVA revealed year × type interaction effects for social jetlag and chronotype, whereas results from the three‐way ANOVAs revealed year × day × type interaction effects for rise time, time in bed, and sleep duration (Table 1). Figure 2 illustrates social jetlag and school day sleep duration in 2019 and 2020, respectively, in relation to circadian typology. The post hoc tests revealed that ΔRise time, ΔTime in bed and ΔSleep duration on schooldays, as well as ΔSocial jetlag were of larger magnitude in evening types compared to intermediate and morning types (Table 2). Chronotype was significantly later in 2020 compared to 2019 in evening types only (*p* < 0.001 paired samples *t* tests), but ΔChronotype did not differ significantly between the circadian types.

**TABLE 2:** Changes in sleep characteristics from 2019 to 2020 (ΔSleep = Sleep 2020 − Sleep 2019) in relation to circadian type

	Circadian typology						Post hoc tests^a^
	Morning (*n* = 194)		Intermediate (*n* = 1065)		Evening (*n* = 744)		
ΔBedtime, hr:min ± min							
Schooldays	00:34 ± 62	*	00:32 ± 65	*	00:41 ± 81	*	
Free days	00:14 ± 78		00:10 ± 76	*	00:10 ± 91	*	
ΔRise time, hr:min ± min							
Schooldays	01:03 ± 74	*	01:26 ± 87	*	01:54 ± 115	*	M ≠ I ≠ E
Free days	00:13 ± 84		00:19 ± 89	*	00:02 ± 95		I ≠ E
ΔTime in bed, hr:min ± min							
Schooldays	00:29 ± 85	*	00:54 ± 96	*	01:13 ± 113	*	M ≠ I ≠ E
Free days	00:00 ± 94		00:08 ± 98	*	−00:08 ± 105		I ≠ E
ΔSleep duration, hr:min ± min							
Schooldays	00:22 ± 90	*	00:39 ± 101	*	01:00 ± 119	*	M, I ≠ E
Free days	−00:04 ± 90		00:07 ± 105		−00:06 ± 111		
ΔSocial jetlag, hr:min ± min	−00:25 ± 70	*	−00:38 ± 66	*	−00:58 ± 82	*	M, I ≠ E
ΔChronotype, hr:min ± min^b^	−00:03 ± 81		00:08 ± 89		00:23 ± 94	*	

All values are presented as mean ± *SD*.

^a^ Comparisons by Tukey HSD post hoc tests. Alpha was set to 0.008.

^b^ Chronotype (MSFsc) could only be calculated for those who did not use an alarm clock on free days, hence these analyses were conducted on 126 morning types, 709 intermediate types and 493 evening types.

* Significant differences from 2019 to 2020 by paired samples *t* test on schooldays and free days separately within each circadian type. Alpha was set to 0.008.

## 4 DISCUSSION

The results from this longitudinal, prospective study showed later rise times, longer school day sleep duration, and reduced social jetlag in adolescent high school students during COVID‐19‐related school lockdown/remote teaching in 2020 compared to pre‐pandemic times in 2019. Furthermore, these changes were of larger magnitude in evening types compared to the other circadian types.

The main difference in sleep from 2019 to 2020 comprised the ~1.5 hr delay in school day rise time. Although also bedtimes were delayed by ~0.5 hr, the adolescents were left with almost 1 hr longer time in bed, providing more opportunity to sleep. Accordingly, mean school day sleep duration increased by 45 min, and the proportion of adolescents who obtained the recommended ≥8 hr of sleep increased by threefold. As sleep is crucial to well‐being, mental and somatic health and daytime functioning (Owens & Weiss, 2017), it is conceivable that the increased sleep duration obtained during school lockdown had positive, and possibly protective, effects on adolescent mental health during the COVID‐19 pandemic. Future studies should address possible associations between sleep changes and mental health during COVID‐19‐related school lockdown, as well as implications for academic performance.

The mean school day rise time in 2020 was as late as 8:25 a.m., which would not be possible under normal circumstances, as schools in Norway usually start between 8:.00 and 8:30 a.m. As such, one may consider the study a naturalistic experiment simulating later school start times. Converging evidence have indicated that later school start times yield more sleep, less daytime sleepiness, improved daytime functioning, and better mental health (Minges & Redeker, 2016; Morgenthaler et al., 2016), but few previous studies have addressed the effects of delaying school start times beyond 8:30 a.m. Results from the present study thus provide novel empirical evidence that school start times even later than 8:30 a.m. may be beneficial with respect to school day sleep duration and social jetlag. Still, these results should be interpreted with caution. Late rise times during the COVID‐19 pandemic likely reflect the combined impact of delayed/flexible school start times, elimination of commuting, less time used for personal needs, oversleeping and/or school absence, and may not necessarily be comparable to late rise times due to late school start times under normal conditions. Furthermore, it is of note that during school lockdown in 2020, the mean rise time was only 11 min prior to the mean start time for remote teaching. Thus it appears that many adolescents attended remote classes directly upon rising (or even in bed), not spending time on breakfast or personal needs. One might worry that such behaviours may negatively affect health, well‐being and learning outcomes, and we recommend this to be addressed in future studies.

In terms of circadian typology, adolescents in the present study were classified based on a questionnaire for circadian preference (rMEQ) (Adan & Almirall, 1991; Horne & Ostberg, 1976) completed in phase 1 (2019). Circadian preference is assumed to represent a relatively stable measure of circadian typology, although such preference generally changes with age (Randler et al., 2017). The positive effects of school lockdown appeared to be largest in adolescents with an evening preference (evening types), who were the ones with the latest and shortest sleep in 2019 (Figure 2). Thus, it appears that potential positive effects of school lockdown in terms of sleep, increase with the degree of eveningness. Similar findings have been reported in a slightly older student sample, showing that those with shortest sleep duration prior to the pandemic experienced the largest increase in sleep during school lockdown (Wright et al., 2020).

It is crucial to stress that the impact of school lockdown on sleep is likely to be present only for the duration of the lockdown period. Whenever normal daily routines are resumed (i.e. re‐opening of schools), the adolescents may slip back into old sleep patterns. They may even be worse off, as suggested by recent evidence that delayed sleep and reduced social jetlag in a large Argentinian sample were accompanied by a delayed chronotype (Leone et al., 2020). Chronotype as a concept is related to circadian typology, but reflects behaviour rather than preference (usually measured as MSFsc), and is thus more likely to change rapidly according to social demands (Leone et al., 2020; Roenneberg et al., 2019). In the present study, after 50–60 days of school lockdown/remote classes, there was only a slight delay in chronotype of ~12 min (small effect size), and free day sleep duration was not changed compared to 2019. Thus, school lockdown appeared to promote school day sleep with only minor effects on the adolescents’ biological timing for sleep. Still, future studies should investigate whether and how re‐opening of schools impacts adolescent sleep patterns. It may be useful to consider gradually earlier school start times, to allow the adolescents time to adjust to the earlier social obligations.

To the best of our knowledge, this is the first large scale study specifically addressing sleep patterns prior to and during COVID‐19‐related school lockdown in adolescent high school students, and the first to explore associations with circadian typology. Studies addressing sleep patterns in older or more diverse samples have yielded similar results in terms of delayed sleep timing and more time in bed during the pandemic (Cellini et al., 2020; Korman et al., 2020; Leone et al., 2020; Wright et al., 2020), but also poorer sleep quality, reduced sleep efficiency, and increased sleep problems (Benham, 2020; Cellini et al., 2020). A combination of increased difficulties initiating sleep and longer school day sleep duration was also reported by Becker et al., in their prospective study among a small sample of adolescents (Becker et al., 2021). Thus, the COVID‐19‐related school lockdown may seem to represent a double‐edged sword, where some perish, and others prosper from loss of daily routines and social demands. Following this line of reasoning it seems important that future studies explore whether (and which) sleep markers can be useful when seeking to identify adolescents at risk for negative health outcomes in relation to the COVID‐19 pandemic and associated social restrictions.

### 4.1 Strengths and limitations

The strengths of the present study include its prospective longitudinal design and the relatively large, population‐based sample, as well as the use of validated questionnaires to address sleep and circadian typology. Moreover, both survey phases were administered at approximately the same time of year (spring), thereby reducing the possible impact of season on sleep. Another strength is that the questionnaire allowed us to account for wakefulness in bed and thus compute actual time spent asleep, according to self‐report.

Still, the present study has some limitations that should be considered when interpreting the data. First, in terms of representativeness, participation in phase 1 was facilitated by the schools, allocating 1 school hour to respond the questionnaire, whereas participants in phase 2 were required to respond to the questionnaire in their spare time. This procedure yielded a response rate in phase 2 of ~60%. Participation in phase 2 appeared slightly biased, with responders being slightly younger, more often female, and fewer had mothers with university/college education. Responders were also less likely to be evening types, and they had longer school day sleep duration and less social jetlag at phase 1 compared to the non‐responders. Second, the present study aimed exclusively to explore the impact of school lockdown on sleep habits, as measured by self‐report. Other aspects of sleep, such as sleep quality, sleep continuity, daytime vigilance, and sleep‐related behaviours, as well as possible effects on sleep and mental health problems were not addressed. Moreover, the present study did not report data on daytime sleep, which may have changed during lockdown as a consequence of staying in bed, being less active, having fewer social activities or due to increase in nocturnal sleep. Thus, future studies are needed to address the impact of the COVID‐19 pandemic on these parameters.

Further, being an unexpected, naturalistic experiment due to the rise of the SARS‐CoV‐2 pandemic, we have no control group allowing us to control for the effect of age (all participants were 1 year older during phase 2, which may have implications for sleep). However, it is unlikely that age can account for a mean rise time at 8:25 a.m. on schooldays (as school normally starts around 8:30 a.m.), thus we assume that the major changes in sleep from 2019 to 2020 were caused by the COVID‐19 pandemic and associated social restrictions. Moreover, we infer that changes that affect school day sleep specifically are likely to be caused by school lockdown, as other possible COVID‐19‐related changes in sleep (e.g. due to worry, stress, or illness) would likely affect school day and free day sleep similarly.

## 5 CONCLUSION

During the initial phase of the COVID‐19 pandemic, when schools were in lockdown, adolescents had later school day rise times, obtained longer sleep duration on schooldays, and social jetlag was reduced. Effects were larger among evening type adolescents. These findings may not only have relevance for our understanding of the impact of the COVID‐19 pandemic and associated social restrictions on adolescents’ sleep and mental health, but also for the debate concerning later school start times for adolescents.

## CONFLICT OF INTEREST

The authors have no conflict of interest to disclose.

## AUTHOR CONTRIBUTIONS

All authors contributed substantially to the conception/design of the work, or the analysis or interpretation of the data. Furthermore, all authors drafted or revised the paper, and approved the final version of the paper.

## Data Availability

Data may be provided upon request.
